# Lenalidomide in heavily pretreated refractory diffuse large B-cell lymphoma: a case report

**DOI:** 10.1186/1752-1947-8-325

**Published:** 2014-10-02

**Authors:** Katarzyna Krawczyk, Wojciech Jurczak, Krystyna Gałązka, Andrzej Gruchała, Aleksander B Skotnicki

**Affiliations:** 1 Department of Hematology, Jagiellonian University, Kopernika 17, Kraków 30-501, Poland; 2 Department of Pathology, Jagiellonian University, Kraków, Poland; 3 Department of Tumor Pathology, Center of Oncology, M. Sklodowska-Curie Memorial Institute, Cracow Branch, Kraków, Poland

**Keywords:** Chemotherapy, Diffuse large B-cell lymphoma, Lenalidomide, Radioimmunotherapy, Rituximab

## Abstract

**Introduction:**

In diffuse large B-cell lymphoma, first-line treatment with rituximab, cyclophosphamide, doxorubicin, vincristine and prednisone; salvage with cisplatin-based regimens for relapsing patients; and autologous stem cell therapy are standards of care. Treatment approaches are less clear for patients who are refractory or who are not candidates for autologous stem cell therapy. Options may include palliative regimens or clinical trial enrollment. One therapy under investigation in diffuse large B-cell lymphoma is lenalidomide, an immunomodulatory agent with antiangiogenic activity.

**Case presentation:**

We present the case of a 55-year-old Caucasian male patient diagnosed with diffuse large B-cell lymphoma who had an early relapse after treatment with rituximab, cyclophosphamide, doxorubicin, vincristine, and prednisone. He then had a subsequent early relapse after cisplatin-based salvage consolidated with autologous stem cell therapy. The efficacy of gemcitabine-cisplatin-rituximab was limited to five months, followed by systemic and central nervous system progression. Fourth-line treatment with lenalidomide plus rituximab and involved-field radiotherapy followed by lenalidomide monotherapy greatly improved this patient’s quality of life and performance status, allowing over two years of progression-free survival to date (excluding a brief relapse due to treatment interruption).

**Conclusion:**

A lenalidomide-based regimen was highly effective in this patient with diffuse large B-cell lymphoma.

## Introduction

Although diffuse large B-cell lymphoma (DLBCL) is an aggressive lymphoma, immunochemotherapy with rituximab, cyclophosphamide, doxorubicin, vincristine, and prednisone (R-CHOP) - widely regarded as the standard of care for first-line patients [1,2] - is effective in more than 50% of cases [3]. Treatment is not as defined for patients who are refractory or who relapse following standard first-line therapy. A number of agents are currently in development for treating relapsed or refractory DLBCL.

Lenalidomide (Revlimid®, Celgene Corporation, Summit, New Jersey, USA) is an immunomodulatory agent that is approved for patients with previously treated multiple myeloma and for patients with myelodysplastic syndrome who have the 5q cytogenetic abnormality. Lenalidomide has both direct tumoricidal and immunomodulatory effects. Direct effects include inhibition of vascular endothelial growth factor-mediated microvessel formation, indicating antiangiogenic and antimetastatic activities [4,5], as well as inhibition of nuclear factor kappa B to bring about cell cycle arrest and tumor cell death [6]. Immunomodulatory effects of lenalidomide include inhibition of pro-inflammatory cytokines such as tumor necrosis factor α, increased anti-inflammatory cytokines such as interleukin-10, increased cytotoxicity of natural killer (NK) cells, and inhibition of regulatory T cells [7-11]. In addition, lenalidomide is a potent enhancer of NK cell-mediated and monocyte-mediated tumor cell antibody-dependent cellular cytotoxicity in non-Hodgkin’s lymphoma cells treated with the anti-CD20 monoclonal antibody rituximab [12].

Numerous studies of lenalidomide in DLBCL as monotherapy or in combination with other agents are ongoing in first-line patients and relapsed or refractory patients, as well as in the maintenance setting. In first-line patients with DLBCL, the combination of lenalidomide with rituximab plus CHOP (R2-CHOP) has yielded promising results in phase 1 and 2 studies [13-18]. In relapsed or refractory DLBCL, data from phase 2 studies of lenalidomide monotherapy and combination therapy with rituximab have been presented [19-24], and overall or objective response rates range from 28% (DLBCL subset) [20] to 35% [21] in this setting. Based on presumptive cell of origin, there are two primary DLBCL subtypes with distinct pathophysiology: germinal center B-cell (GCB) and activated B-cell (ABC)/non-GCB lymphoma. These are associated with different prognoses. The ABC/non-GCB subtype has a significantly poorer prognosis than the GCB subtype, and this correlation is independent of the International Prognostic Index (IPI) [25,26]. The benefit of lenalidomide may differ depending on the subtype of DLBCL [14,27]. In patients with relapsed or refractory DLBCL treated with salvage lenalidomide, a higher overall response rate (ORR) has been observed in patients with the non-GCB subtype compared with those with the GCB subtype (ORR 52.9% versus 8.7%; *P*=0.006) [27]. The benefit of lenalidomide has also been reported in newly diagnosed patients with non-GCB subtype DLBCL treated with R2-CHOP. In this setting, several small (<70 patients), phase 2 studies have demonstrated that the addition of lenalidomide improves progression-free survival and overcomes the negative prognostic impact of the non-GCB subtype on patient outcome [14,18]. An ongoing phase 2 randomized trial (NCT01856192) is comparing progression-free survival in patients with GCB and non-GCB DLBCL treated with first-line lenalidomide combined with R-CHOP versus R-CHOP alone [28]. *In vitro* studies indicate that the differential effects of lenalidomide on non-GCB DLBCL cells are dependent on the expression of interferon regulatory factor 4 and cereblon, an E3 ubiquitin ligase complex co-receptor protein [29].

Prognosis is poor for patients with multiply relapsed or refractory DLBCL, and current treatment guidelines suggest either autologous stem cell transplant (ASCT) or treatment within a clinical trial [1,2], underscoring the dearth of therapeutic options for this population. In this report, we present the case of a patient with DLBCL who received lenalidomide plus rituximab after multiple relapses on other treatments.

## Case presentation

**Figure 1:**
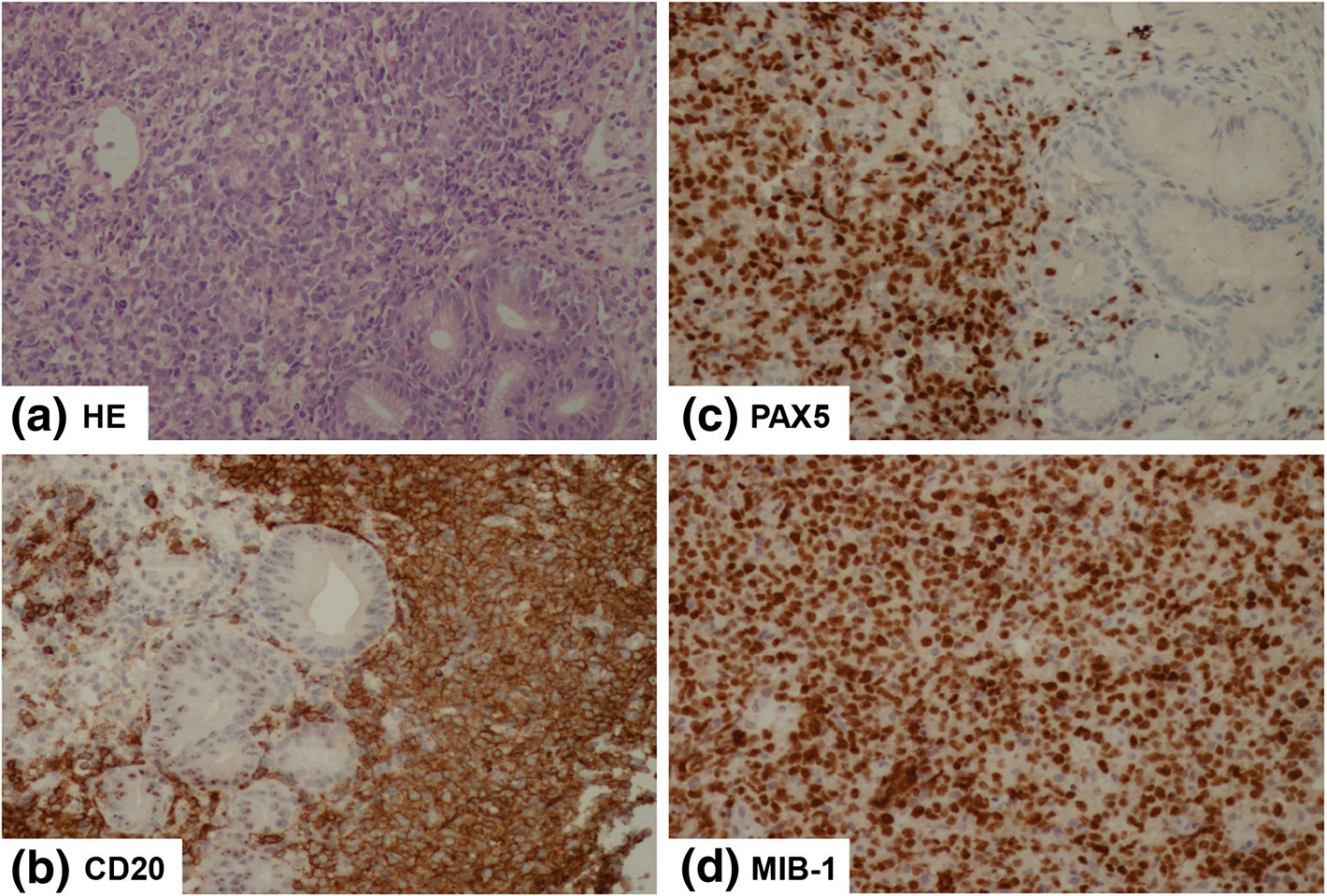
**Initial histopathologic examination of the gastric biopsy specimen. (a)** Gastric mucosa occupied (the left two-thirds of the image) by the diffuse infiltrate composed of large lymphoma cells. Hematoxylin and eosin (HE) stain, objective magnification 40×. **(b)** CD20 stain positivity in lymphoma cells, objective magnification 40×. **(c)** PAX5 stain positivity in lymphoma cells, objective magnification 40×. **(d)** High proliferative activity of diffuse large B-cell lymphoma cells on immunohistochemical staining for MIB-1 (Ki67), objective magnification 40×.

Our 55-year-old Caucasian male patient presented with a two-month history of gastrointestinal symptoms, including severe dyspepsia, vomiting, and ‘black stool.’ At that time, it was not clear whether our patient’s weight loss (14kg), high fever (>38.5°C), and drenching night sweats were general symptoms or whether they were the result of an undiagnosed gastrointestinal tract disorder. The first histopathological confirmation of lymphoma came from a gastric biopsy sample (Figure 1) that confirmed CD20-positive DLBCL with a high proliferation fraction (expressed by Ki67 immunohistochemical stain, which was positive in 90% of the cells). Furthermore, the cells were positive for MUM1 and negative for CD5 by immunohistochemistry. Our patient was referred from primary care to a hematologist following analysis of the gastric biopsy sample. By the time imaging studies were arranged (one week), generalized lymphadenopathy was evident, including a cervical lymph node that was nearly 8cm in diameter. Positron emission and computed tomography (PET-CT) imaging demonstrated Ann Arbor stage IVB with enlarged submandibular, cervical, and mediastinal lymph nodes; lung and spleen nodules (172mm); and gastric infiltration. At the beginning of therapy, our patient had a high IPI score of 4.

**Figure 2:**
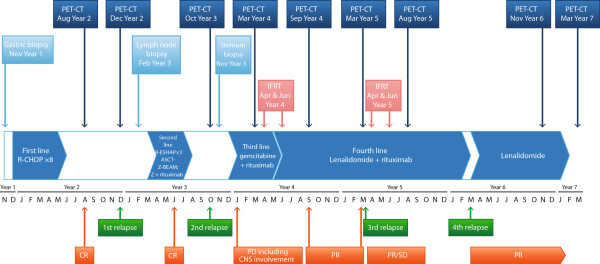
**Treatment schema.** ASCT-Z-BEAM, autologous stem cell transplant conditioned by Z-BEAM (Zevalin® and carmustine, etoposide, cytarabine, and melphalan); CNS, central nervous system; CR, complete response; IFRT, involved-field radiotherapy; PD, progressive disease; PET-CT, positron emission tomography-computed tomography; PR: partial response; R-CHOP: rituximab plus cyclophosphamide, doxorubicin, vincristine, and prednisone; R-ESHAP: rituximab plus etoposide, methylprednisolone, cisplatin, and cytarabine; SD: stable disease; Z: Zevalin®.

In the first year of therapy (year 1), our patient was treated with standard R-CHOP chemotherapy (Table 1) from December of year 1 through May of year 2. A good clinical response was confirmed on CT imaging performed after cycles four and eight (treatment schema, Figure 2). Three months after completing first-line therapy, in August of year 2, a complete response (CR) status was further confirmed by a PET-CT scan that revealed cervical lymph nodes (<20mm in the long axis), with a standard uptake value (SUV) of 2.1, which was below mediastinal blood pool structure (MBPS) and liver maximum SUVs (SUVmax) and one 5mm nodule in the fifth segment of his left lung.

**Table 1:** Treatment regimen for a heavily pretreated patient with diffuse large B-cell lymphoma

**First-line treatment**	
R-CHOP×8	Rituximab 375mg/m^2^ (D1)
	Cyclophosphamide 750mg/m^2^ (D1)
	Doxorubicin 50mg/m^2^ (D1)
	Vincristine 1.4mg/m^2^ (D1)^a^
	Prednisone 40mg/m^2^ (D1 to D5)
**Second-line treatment**	
R-ESHAP×3	Rituximab 375mg/m^2^ (D1)
	Etoposide 40mg/m^2^/day (D1 to D4)
	Methylprednisolone 500mg/m^2^/day (D1 to D4)^b^
	Cisplatin 25mg/m^2^/day (D1 to D4)^c^
	Cytarabine 2000mg/m^2^ (D5)
Z-BEAM	Y90-labeled ibritumomab tiuxetan (ZEVALIN)
	Carmustine
	Etoposide
	Cytarabine
	Melphalan
	Followed by rituximab 375mg/m^2^, followed by Y90-labeled ibritumomab tiuxetan 32mCiu/kg and BEAM (carmustine, etoposide, cytarabine, melphalan) -conditioned autologous stem cell transplant
**Third-line treatment**	
	Gemcitabine 1000mg/m^2^ (D1 to D5)
	Cisplatin 80mg/m^2^ (D1 to D5)
	Rituximab 375mg/m^2^ (D1 to D5)
	Prednisone 60mg/m^2^ (D1 to D5)
	Followed by gemcitabine 1,000mg/m^2^ (every two weeks) and rituximab 375mg/m^2^ (every four weeks)
Intravenous steroids	Dexamethasone 3×8mg
	Liposomal cytarabine 50mg (two doses)
Radiotherapy	Involved-field RT and prophylactic RT (26Gy per 10 fractions), then involved-field RT (8Gy per 1 fraction)
**Fourth-line treatment**	
	Lenalidomide 25mg
	Rituximab 375mg/m^2^ (every four weeks)
	Palliative RT
	Palliative RT
	Lenalidomide 10mg/day for 21 days (every four weeks)

^a^Maximum 2mg/dose; ^b^maximum 1,000mg/dose; ^c^continuous 24-hour infusion. D, day; Gy, gray; RT, radiotherapy.

Our patient’s quality of life during first-line treatment was rather good; symptoms disappeared quickly. For the most part, he participated in normal daily activities. Treatment was conducted primarily in an outpatient setting.

In December of year 2, CT assessment suggested possible progression in cervical lymphadenopathy; early relapse was eventually confirmed by histopathology seven months after completion of the first-line therapy. As a second line of therapy, our patient received a regimen of rituximab, etoposide, methylprednisolone, cisplatin, and cytarabine (R-ESHAP) (Table 1) from March of year 3 to May of year 3.

**Figure 3:**
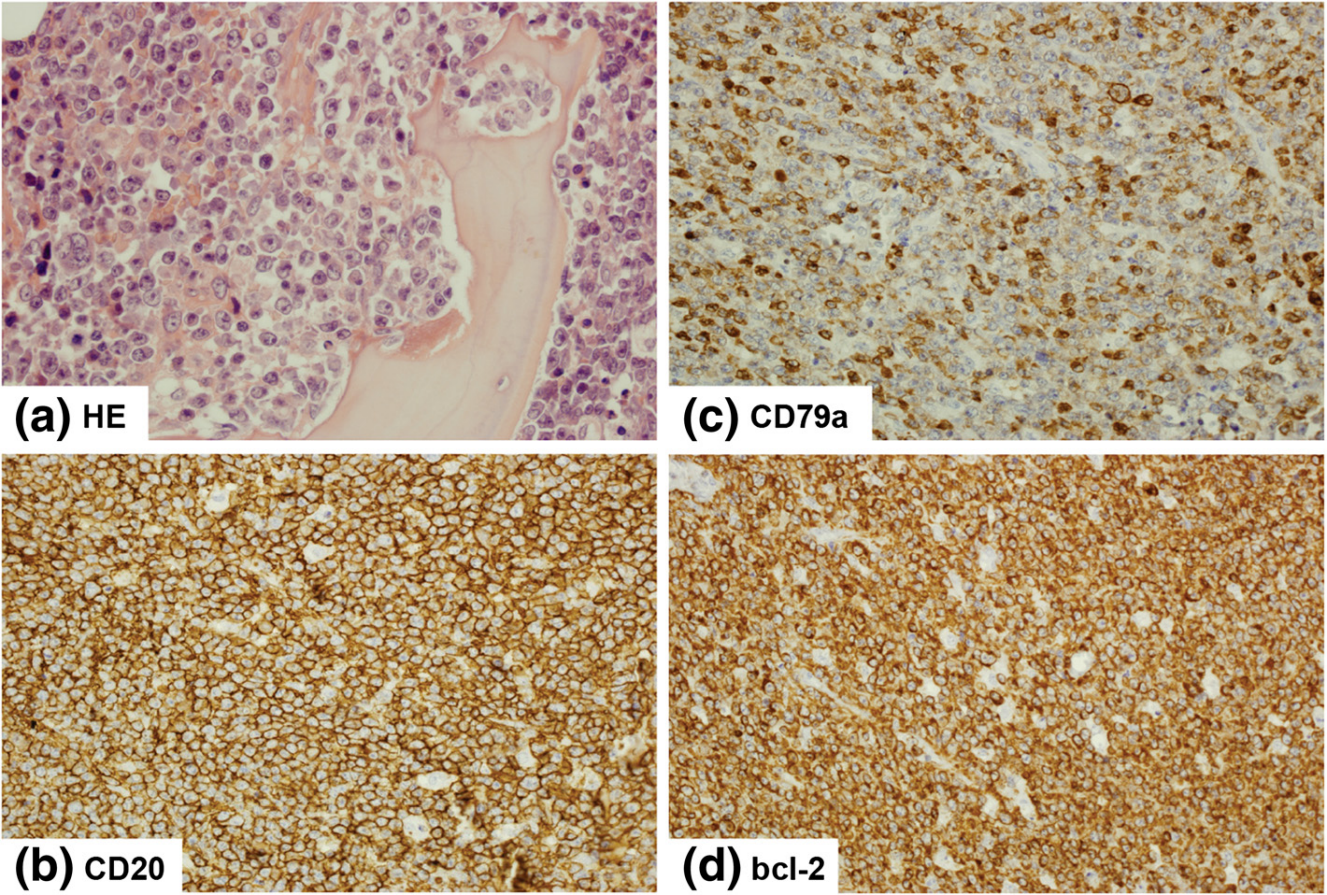
**Relapse in the sternum. (a)** A bone trabecula in the section of the sternal bone infiltrated and destroyed by diffuse large B-cell lymphoma (DLBCL) infiltrate. Hematoxylin and eosin (HE) stain, objective magnification 60×. **(b)** Strong expression of CD20 in the DLBCL cells, objective magnification 40×. **(c)** CD79a expression varying in intensity in some cells of the neoplastic infiltrate, objective magnification 40×. **(d)** Strong expression of the Bcl-2 protein in the DLBCL infiltrate, objective magnification 40×.

A second CR was further consolidated by Z-BEAM-conditioned (Y90-labeled ibritumomab tiuxetan (Zevalin®, Spectrum Pharmaceuticals, Henderson, Nevada, USA) combined with the carmustine, etoposide, cytarabine, and melphalan regimen (BEAM)) ASCT. An initial dose of rituximab in June of year 3 was followed a week later by a second rituximab dose plus Y90-labeled ibritumomab tiuxetan and BEAM-conditioned ASCT 11 days after initiating therapy. Our patient was released from the hospital in July of year 3, with good hematopoietic reconstitution and no complications.At a three-month follow-up visit in October of year 3, our patient presented with no symptoms or abnormalities on physical examination. However, a small lesion was detected by PET-CT in his sternum (12mm in diameter, SUVmax 22.2, MBPS 3.2, liver SUVmax 3.8). A relapse of his DLBCL with high proliferation fraction was confirmed in material from an urgent partial sternectomy (Figure 3). Despite this prompt procedure performed by thoracic surgeons, the disease progressed in the following months, as multiple bone lesions were found on subsequent PET imaging.

Our patient’s quality of life during second-line treatment and ASCT was poor. There were extensive inpatient periods and side effects from the intensive chemotherapy. Furthermore, his survival concerns caused great stress and discomfort.

In December of year 3, our patient started third-line, gemcitabine-based chemotherapy for debulking (Table 1). He received gemcitabine with cisplatin, rituximab, and prednisone. This was followed by gemcitabine every two weeks and rituximab every four weeks.

Beginning in January of year 4, our patient began to present unspecified, transient symptoms suggestive of central nervous system (CNS) involvement: headaches and signs of VII cranial nerve paralysis. Although this conclusion could not be definitively diagnosed by lumbar puncture with cerebrospinal fluid (CSF) cytology and repeated imaging studies (CT and magnetic resonance imaging), intrathecal liposomal cytarabine was given as CNS prophylaxis. In April of year 4, our patient presented with diplopia and right-side ptosis. CT scans demonstrated a left orbit tumor that infiltrated his frontal sinus. A neurological examination and CSF cytology were otherwise normal. Our patient received intravenous steroids and liposomal cytarabine, followed by involved-field radiotherapy (IFRT) of the orbit in April of year 4 and prophylactic radiotherapy of the CNS in May of year 4 (Table 1). The diplopia and ptosis resolved completely, but he developed bone pain in different locations. This was addressed by palliative IFRT in April of year 4.

During this period of treatment, our patient’s quality of life remained poor. He was always either home-bound or hospitalized and was constantly undergoing medical procedures, including lumbar puncture. He experienced pain and the loss of some neurological function, and continued to be in great stress with survival fears.

**Figure 4:**
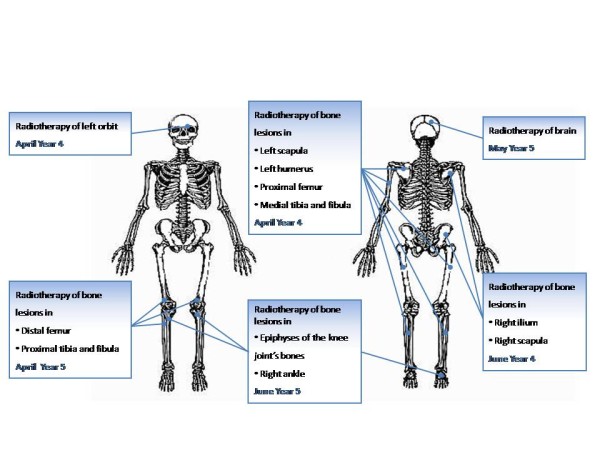
Radiotherapy sites and dates.

**Figure 5:**
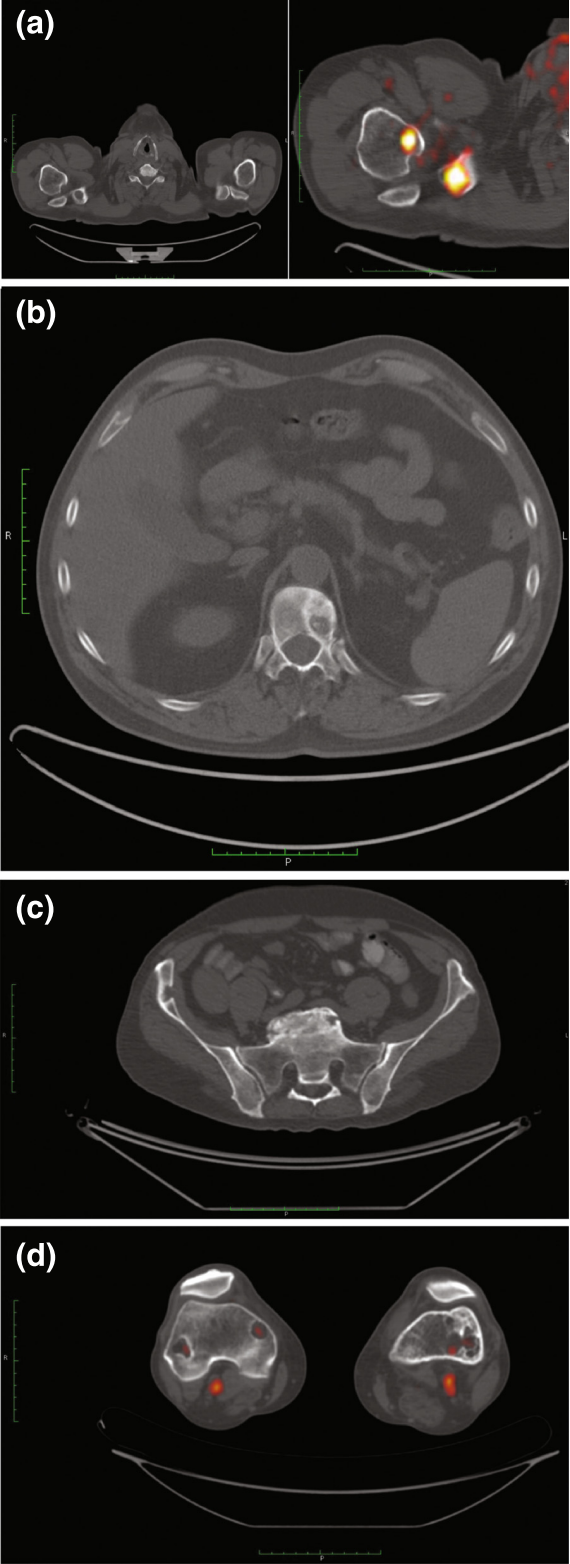
**Positron emission tomography-computed tomography imaging of bone lesions. (a)** Acromial extremity of the right clavicle, March of year 4. **(b)** Thoracic vertebra T12, March of year 4. **(c)** Right ilium, March of year 5. **(d)** Distal extremity of the femur bones, March of year 5.

Our patient remained on the rituximab therapy (375mg/m^2^ every four weeks) initiated during third-line treatment. In May of year 4, he started fourth-line treatment with lenalidomide in addition to rituximab (Table 1). In June of year 4, he underwent a second round of palliative radiotherapy for bone lesions in locations corresponding with bone pain (Figure 4).In September of year 4, PET-CT indicated a partial response; only two bone lesions were found (humeral end of his right clavicle: 17×23mm, SUVmax 7.1; distal and proximal ends of his right tibia: SUV 4.9; MBPS 3.5, liver SUVmax 4.2). Our patient remained free of symptoms until March of year 5, when PET-CT (Figure 5) revealed lesions of increased SUV in his pelvis, bilateral femur and tibia, left ankle, and the humeral end of his right clavicle; SUVmax of these lesions was up to 4.5, MBPS 2.8. An osteolytic lesion in his T12 vertebra was also described. Palliative radiotherapy given in April and June of year 5 was again successful. His pain resolved, and in a PET-CT assessment in August of year 5, no new skeletal lesions were described. The existing lesions were not metabolically active (lesions in his right femur, SUVmax 2.1; left femur, 1.8; bilateral tibias, 1.7 (MBPS 1.9, liver SUVmax 3.2)). The lenalidomide-plus-rituximab combination was continued until February of year 6, when treatment was halted for administrative reasons.

Six weeks after stopping lenalidomide-plus-rituximab therapy, our patient developed palpable lymphadenopathy of 5 to 10mm on his neck, prompting initiation of lenalidomide monotherapy in March of year 6, which has continued to date. Due to grade 3 neutropenia as classified by the Common Terminology Criteria for Adverse Events (version 4.0) [30], the lenalidomide dose was reduced from 25 to 10mg. Since the start of lenalidomide monotherapy, our patient has not experienced any progression, with a negative PET-CT in March of year 7, and has not experienced any bone pain or lesions.

Hematologic adverse events during lenalidomide-based treatment were manageable and comprised transient thrombocytopenia (platelets 39,000 to 80,000/μL, no treatment required), and leuko- and neutropenia (white blood cells <1000/μL, polymorphonuclear cells <500; managed with granulocyte colony-stimulating factor support). The cytopenia was exacerbated during the concomitant palliative radiotherapy. Our patient developed two infections: sinusitis in March of year 5, which was treated with amoxicillin, and herpes (varicella zoster virus infection limited to the skin of his left cheek, with severe neuralgia) from July to October of year 5, which was treated with acyclovir and analgesics.

During treatment with lenalidomide plus rituximab and then later on lenalidomide monotherapy, our patient’s quality of life and performance status remained surprisingly high (World Health Organization grade 0, Karnofsky score 100%) and much improved compared with his quality of life and performance status during previous therapies. Inpatient hospitalization was not required, all procedures were performed in a day clinic, and he returned to full-time work and engaged in physical activities.

## Discussion

DLBCL is the most common aggressive non-Hodgkin’s lymphoma in adults and accounts for 25% to 35% of newly diagnosed cases [3,31]. Despite the significant advances with the addition of rituximab to CHOP chemotherapy [32,33], 30% to 40% of patients are refractory or relapse and eventually succumb. This is particularly frustrating in younger patients with a high proliferation fraction. Attempts to improve results in this subgroup have failed despite early intensification of first-line therapy or consolidation of the first CR with high-dose chemotherapy supported by ASCT [34]. Prognosis for patients with an early relapse after R-CHOP therapy in the first line is particularly poor [35,36], and much worse than previously reported by the PARMA trial [37].

Lenalidomide has an acceptable toxicity profile and is easily manageable and effective in heavily pretreated patients with high-grade lymphoma [38], based on results from four multicenter phase 2 trials by Wiernik *et al*. [19], Witzig *et al*. [20], Zinzani *et al*. [21], and Wang *et al*. [22]. The first two trials explored the efficacy of lenalidomide monotherapy in relapsing or refractory DLBCL. Lenalidomide (25mg for 21 or 28 days) resulted in ORR up to 37% [22] and median progression-free survival up to four months [19,22], with median duration of response from 4.6 to 16 months [20,21]. In the third trial, four cycles of lenalidomide, rituximab, and dexamethasone induction were followed by lenalidomide maintenance [21]. Although the ORR was similar (35%), it was long-lasting in patients receiving lenalidomide maintenance. Toxicity was manageable and well-tolerated, despite the heavily pretreated population. The most frequent grade 3 or 4 adverse events were hematologic (neutropenia 30%, thrombocytopenia 14%, asthenia 5%, and anemia 5%) [21]. These results are confirmed by case reports from everyday practice. For example, Rubenstein *et al*. reported remarkable regression of refractory intraocular DLBCL during monotherapy with lenalidomide [39]. Lenalidomide is not standard treatment for DLBCL at our institution, although we had previously observed positive results among our patients participating in clinical trials of lenalidomide for refractory non-Hodgkin’s lymphoma.

In our patient, we observed an early relapse after first-line R-CHOP chemotherapy, indicating a dynamic and aggressive lymphoma with poor prognosis. Although radioimmunotherapy (ibritumomab tiuxetan) was added to a BEAM conditioning regimen supported by ASCT, it did not overcome disease resistance. Considering the rapid progression of lymphoma at each relapse, it is very unlikely that local, palliative radiotherapy would have effectively cured the disease and prevented distant relapses. Treatment with lenalidomide was considered under compassionate use after failure of all other treatments available at our institution. The lenalidomide-rituximab regimen was based on Wiernik and Witzig protocols [19,20]; however, dexamethasone was omitted to minimize the risk of osteoporosis and compression fractures in a patient with lymphoma-infiltrated vertebrae. This regimen changed the lymphoma dynamics, allowing more effective radiotherapy and possibly preventing its spread to distant sites, and provided the longest progression-free survival (eight months after the R-CHOP chemotherapy and three months after Z-BEAM ASCT versus 12 months on lenalidomide-rituximab until treatment break followed by >12 months during lenalidomide monotherapy). Our patient’s quality of life was excellent and greatly improved compared with previous therapies, treatment was outpatient-based, and he returned to work. Furthermore, this positive clinical response and improved quality of life were maintained despite treatment interruption and continuation as lenalidomide monotherapy for over 10 months.

## Conclusions

We demonstrate that lenalidomide can be efficacious in DLBCL, and we believe that it should be considered in relapsed or refractory DLBCL, particularly for heavily pretreated patients.

## Consent

Written informed consent was obtained from the patient for publication of this case report and accompanying images. A copy of the written consent is available for review by the Editor-in-Chief of this journal.

## Abbreviations

ABC: Activated B-cell; ASCT: Autologous stem cell transplant; BEAM: Carmustine etoposide, cytarabine, and melphalan; CHOP: Cyclophosphamide doxorubicin, vincristine, and prednisone; CNS: Central nervous system; CR: Complete response; CSF: Cerebrospinal fluid; CT: Computed tomography; DLBCL: Diffuse large B-cell lymphoma; GCB: Germinal center B-cell; HE: Hematoxylin and eosin; IFRT: Involved-field radiotherapy; IPI: International Prognostic Index; MBPS: Mediastinal blood pool structures; NK: Natural killer; ORR: Overall response rate; PD: Progressive disease; PET-CT: Positron emission and computed tomography; PR: Partial response; R-CHOP: Rituximab plus cyclophosphamide doxorubicin, vincristine, and prednisone; R-ESHAP: Rituximab plus etoposide methylprednisolone, cisplatin, and cytarabine; SD: Stable disease; SUV: Standard uptake value; SUVmax: Maximum standard uptake value; Z: Zevalin.

## Competing interests

The authors declare that they have no competing interests.

## Authors’ contributions

KK, WJ, KG, AG, and ABS wrote the paper. All authors read and approved the final manuscript.
